# Biomarkers to aid in diagnosis of allergic contact dermatitis

**DOI:** 10.3389/falgy.2025.1564588

**Published:** 2025-02-26

**Authors:** Manuel Sargen, Akimi Sasaki, Anish R. Maskey, Xiu-Min Li

**Affiliations:** ^1^ Department of Pathology, Microbiology, & Immunology, New York Medical College, Valhalla, NY, United States; ^2^ Department of Otolaryngology, New York Medical College, Valhalla, NY, United States; ^3^ Department of Dermatology, New York Medical College, Valhalla, NY, United States

**Keywords:** allergic contact dermatitis, biomarkers, haptens, pathophysiology, diagnosis

## Abstract

Allergic contact dermatitis (ACD) is an increasingly common skin condition characterized by itchy rashes in response to allergens. The most common diagnostic test involves patch testing (PT), but despite the efficacy of PT for identifying and guiding patients toward avoidance of allergens, PT alone does not elucidate the underlying biomechanistic changes which may be useful for sub-categorizing ACD further. In addition, some patients may never be able to identify their causative allergens unless they go to highly specialized ACD centers. Accordingly, this mini review attempts to summarize biomarkers that may help with identifying and sub-categorizing cases of ACD for appropriate diagnosis, especially in patients with difficult-to-identify allergens.

## 1 Introduction

### 1.1 Epidemiology of ACD

Recent epidemiologic studies predict that upwards of 20% of children and adults may be affected by acute or chronic ACD with significant impairments in quality of life (1). One older study has even suggested that 55% of patients studied exhibited signs of ACD to at least one allergen, and the prevalence seems to be increasing (2). The most common allergens in patients with ACD are nickel and other metals, fragrances, and preservatives (3). Additionally, another important allergen associated specifically with ACD is latex, a natural rubber compound found in many products (especially latex gloves). The substance in particular which is believed to be involved in the sensitization of latex are the so-called vulcanization accelerants which polymerize the latex into sheets which can be made into industrial products (4). In one study, between 5.4% and 7.6% of the general population were found to be sensitized to latex (5). This number increases to 10%–20% when healthcare workers are studied independently (5). Even though ACD to latex is common, it must be distinguished from irritant contact dermatitis, which may be even more common than ACD in occupational settings (6). ACD is also twice as likely to occur in women and can often be seen in children and adolescents (3). Occupational contact dermatitis is, in many countries, the leading occupational disease, with an estimated incidence rate around 0.5–1.9 cases per 1,000 full-time workers per year (7). Although common in all groups, genes, age, sex, and ethnicity are among the main risk factors for susceptibility for ACD (3).

### 1.2 Clinical presentation of ACD

Most often, the clinical presentation of ACD begins as an eczematous process characterized by pruritus, erythema, edema, vesicles, and crusting. In some patients, however, a non-eczematous subtype may be present characterized by predominantly urticarial, granulomatous, acneiform, lichen planus-like, or dry, hyperkeratotic lesions (8). These different clinical presentations may make it difficult for some clinicians to differentiate between ACD and similar skin conditions such as atopic dermatitis and irritant contact dermatitis—a similar process to ACD, but without an allergic immune response. However, more attention is being brought to the different variations of eczema and eczema-like skin conditions such as ACD, yielding helpful results for the differentiation of each condition.

### 1.3 Pathophysiology of contact dermatitis

Despite classically being defined as a Type IV hypersensitivity reaction, both Type I and Type IV hypersensitivity reactions can be seen in ACD cases, sometimes simultaneously and sometimes sequentially (9).

**Figure 1:**
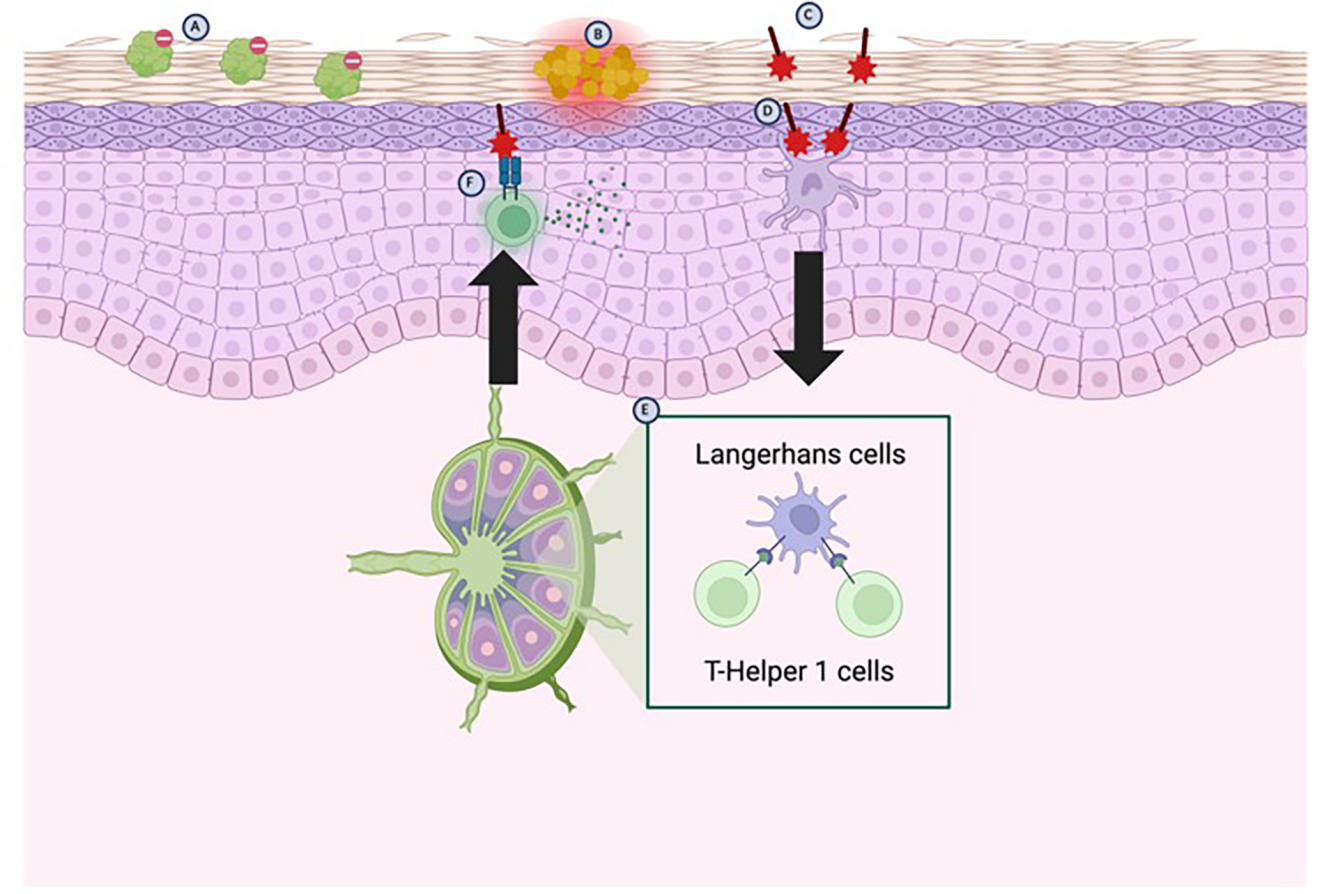
Pathophysiology of ACD. Genetic absence or loss-of-function mutations in the FLG (filaggrin) gene predisposes patients to a disrupted skin barrier **(A)** The weakened skin barrier is then susceptible to colonization by *Staphylococcus aureus* **(B)** displaces commensal skin bacteria, which further weakens the skin barrier by releasing products which inhibit fatty acid elongation, leading to an accumulation of shortened fatty acids. The further weakened skin barrier then allows for haptens to enter and bind to endogenous proteins in the skin **(C)** which are then detected mainly by Langerhans cells, but to some degree by resident dendritic cells **(D)** The Langerhans cells migrate to local lymph nodes where they activate T-Helper 1 cells **(E)** T-Helper 1 cells then migrate back to local tissues and upon re-exposure to the antigens trigger release of pro-inflammatory cytokines **(F)** (created with BioRender).

Sensitization to an allergen begins with conversion of pro-haptens to haptens, a process which depends on keratinocytes for enzymes to facilitate the conversion (10). Immunologically active haptens are then formed after inactive haptens penetrate the stratum corneum and covalently bind to endogenous proteins and trigger an immune response (10, 11) (Figure 1C). It is thought that pre-existing skin barrier dysfunction is necessary for antigens to penetrate the stratum corneum and thus trigger sensitization (10) (Figures 1A,B). Once the process of sensitization has begun, keratinocytes encountering the now-immunogenic antigen triggers keratinocyte release of inflammatory molecules which are responsible for the classical symptoms associated with ACD (10).

Following the innate immune response, local antigen-presenting cells (mainly Langerhans cells, but dendritic cells as well) migrate to regional lymph nodes and activate antigen-specific T-cells (Figures 1D,E); the predominant activation of T-helper 1 (T_H_1) cells results in the classic Type IV hypersensitivity reactions associated with ACD (12) (Figure 1F). These antigen-specific T-cells now enter the circulation and the site of original exposure, such that re-exposure to the antigen triggers activation of T-cells via cytokines and induces an inflammatory response (12) (Figure 1F).

### 1.4 Current measures for diagnosis and screening

ACD is usually first suspected in patients who have rashes in distributions that would be suggestive of frequent contact with allergens (12). One commonly seen distribution is on the lower abdomen due to frequent contact with nickel belt buckles. Other areas which are common distributions of ACD include hands, feet, face, and eyelid, as well as unilateral presentations (12). Another indication by which patients should undergo PT is those who present with rashes and work in occupations with frequent use of chemicals or irritants that are commonly linked to ACD (12). Currently, a diagnosis of ACD is made by screening through a complete history that leads clinicians to suspect ACD followed by confirmatory PT.

PT is done by occluding allergens at concentrations below what would typically be irritating for patients without ACD on the patient's skin for 2 days, allowing it time to develop an immune response that can be seen and measured, while also allowing enough time for transient inflammatory responses to subside in patients without ACD (12, 13). However, even after an unremarkable 48-h incubation, re-testing or delaying reading by an additional 24–48 h may be necessary in some patients (12). Many patients may struggle with itching at the sites of PT, and it may also be difficult for some patients to avoid getting the testing sites wet for the 48-h incubation period.

## 2 Biomarkers for diagnosis

Current screening and diagnostic measures for ACD may be unreliable and can sometimes be unable to distinguish between equally common conditions that may appear similar, such as atopic dermatitis and irritant contact dermatitis. To find more objective measures that can lead clinicians to identify and diagnose ACD, we have compiled biomarkers present in patients with ACD for this purpose.

Biomarkers that can be used to distinguish ACD from other similar skin conditions for diagnostic purposes can be divided into four categories: indicators of skin barrier dysfunction, biomarkers indicating that immunologically active haptens are being formed, cytokines and other inflammatory markers, and genetic markers that may make individuals susceptible to sensitization. In the following sections, we will discuss these biomarkers within their corresponding categories.

### 2.1 Skin barrier dysfunction

The first step in sensitization usually involves dysfunction of the skin barrier such that it cannot prevent antigens from penetrating the stratum corneum. Skin barrier integrity often becomes compromised in the context of skin microflora dysbiosis. Commensal bacteria on the surface of the skin provide a protective benefit by secreting antimicrobial peptides (14). Loss of these bacteria on the surface of the skin results in withdrawal of the protective antimicrobial peptides, which then allows *Staphylococcus aureus* to colonize the surface of the skin (14) (Figure 1B). Recent studies have shown that *S. aureus* colonization causes aberrant epidermal lipid compositions, which in turn results in skin barrier dysfunction (15). Changes in lipid composition involve accumulation of shorter length fatty acids by downregulating the enzymes responsible for creating longer fatty acid chains—known as elongases (15). Accumulation of 16–18 carbon long fatty acids is most associated with skin barrier dysfunction resulting from *S. aureus* colonization and these lipid changes are especially significant when the skin is colonized by MRSA strains (15). As a result of these changes, lipid analysis suggests a fatty acid shortening on the skin as well as cultures positive for *S. aureus* may be indicative of ACD but may also include other differentials.

### 2.2 Indicators of hapten formation and activation

Protein-hapten binding occurs after protein-reactive chemicals act as sensitizers through a process known as haptenation (16) (Figure 1C). Theoretically, protein-bound haptens could be isolated and identified. However, this is not currently done for ACD *in vivo* besides for research purposes despite protein-hapten binding being an integral part of the pathogenesis of ACD. In addition to protein-bound haptens being identified *in vivo*, certain molecules such as glutathione may also provide some basis for suspecting protein-bound haptens in the skin. In one study, 13/14 sensitizers were able to bind glutathione due to its thiol group and, moreover, glutathione is a common endogenous peptide which is used for detoxification (16). As a result of its detoxifying effect and ability to bind haptens, glutathione can be depleted and in patients with ACD a lower concentration of glutathione may be expected than in unaffected skin (16).

### 2.3 Cytokines and other inflammatory markers

The most clinically impactful biomarkers for ACD are likely to be cytokines and other inflammatory markers associated with the disease. In fact, the symptomatology of ACD is unlikely to occur without the downstream effects of these inflammatory molecules secreted by the activated immune system. Once sensitization has occurred at the local level, alarmins and cytokines are secreted by the keratinocytes activated by protein-bound haptens. Alarmins have downstream effects to activate toll-like receptors (TLRs) such as TLR2 and TLR4; TLR2 and TLR4 have further effects to activate nuclear factor-ĸB-dependent proinflammatory cytokines such as IL-1a, IL-1b, TNF-α, granulocyte-macrophage colony-stimulating factor, IL-8 and IL-18 (10). IL-1a is mainly responsible for the induction of skin sensitization to antigen, whereas IL-1b is required for Langerhans cell migration (Figure 1D). The products of the keratinocytes subsequently act to activate innate immunity and activate the T-cell response characteristic of Type IV hypersensitivity reactions (Figures 1E,F). Some cytokines associated with downstream pathways of ACD may also be helpful in identifying ACD lesions. One study, for instance, identified that IL-31—a cytokine associated released by activated T_H_2 cells—was present in skin lesions of patients with atopic dermatitis and ACD, but not psoriasis (17). Despite the strong association of these inflammatory markers with ACD, however, they remain non-specific markers present in other atopic conditions such as atopic dermatitis and so they may not be helpful in aiding diagnosis of ACD exclusively (10, 12, 17).

### 2.4 Genetic biomarkers

Polymorphisms in the FLG gene—the gene coding for the filaggrin protein—have been shown to predispose patients to having a dysfunctional skin barrier that may allow for sensitization by chemicals and antigens (17) (Figure 1A). Indeed, a loss-of-function mutation or deficiency in the FLG gene has been well-characterized as the strongest known genetic risk factor for skin barrier dysfunction in atopic dermatitis (14). Lack of integrity of the skin barrier due to lack of functional copies of filaggrin allow haptens to penetrate the stratum corneum and lead to sensitization of the skin towards the antigen while also providing a point of entry for *S. aureus* to colonize the skin and potentiate the inflammatory response in response to the antigen.

In some rare cases of ACD, some antigens may directly sensitize the skin, bypassing the innate immune response. One of the proposed mechanisms by which these so-called contact sensitizers produce an ACD response is by covalently binding to cysteine residues on a cytosolic protein called Keap1. This protein is typically a sensor for oxidative and electrophilic stress, which degrades Nrf2—an intracellular transcription factor—by proteasomal degradation, but these covalent modifications prevent it Keap1 from ubiquitinylating Nrf2. Nrf2 is then free to transcriptionally promote antioxidant changes in the cell, protecting them from inflammatory effects. Knockout studies in mice without Nrf2 have shown that mice lacking Nrf2 become sensitized with antigens that typically do not sensitize in wild-type mice (10), indicating a possible genetic basis for susceptibility for ACD to develop.

Other studies have suggested polymorphisms and mutations in genes coding for interleukins may predispose some patients to developing ACD, but these have not been well-characterized and may require more research before they can be used for diagnostic purposes. One study identified an association between IL-16 polymorphisms and ACD in patients who are sensitized to one or more allergens (18). However, in-depth analyses of interleukins and polymorphisms that may be responsible for a genetic basis of ACD, are not diagnostic on their own since there is an overlap with other atopic conditions.

## 3 Discussion

ACD is generally felt to be underdiagnosed for several reasons, including difficulty with correctly diagnosing and differentiating it from similar conditions such as atopic dermatitis, lichen planus, or angioedema (19). Other obstacles for diagnosis of ACD include low proportion of patients seeing clinicians who can provide them with a diagnosis since many patients opt to forego treatment for dermatologic conditions (19). The most important distinguishing feature between ACD and atopic dermatitis is the presence of symptomatic skin at sites which may come in contact with allergens. However, systemic absorption of allergen and movement of allergens from one part of the body to another may result in ACD rashes on distant sites which may be difficult to identify as ACD over atopic dermatitis (19). While eczematous lesions on the skin are the most common symptom of ACD, some patients may experience distinct manifestations which are not commonly associated with atopic dermatitis, such as: erythema multiforme, lichen planus, eruptive rashes, and pigment changes (19).

Currently, there are gaps in our knowledge of the pathogenesis of ACD and how it may differ from other allergic conditions and other skin conditions such as atopic dermatitis and psoriasis. Because of this, it is difficult to use biomarkers to differentiate skin lesions as ACD from other similar-appearing conditions which may be prone to irritation by PT. As a result, we conclude that there needs to be more research done to fill in the gaps when it comes to biomarkers that may be pathognomonic for ACD. One example discussed in this paper is how skin barrier dysfunction is usually considered a prerequisite for ACD to develop, but it is also a non-specific process that has been tied to several other conditions. Indeed, skin barrier dysfunction has been linked with atopic dermatitis, childhood asthma, food allergy, and allergic rhinosinusitis (17). As a result of this, while we have examined some biomarkers suggesting skin barrier dysfunction in patients with ACD, we do not expect biomarkers of skin barrier dysfunction alone to provide a basis for the diagnosis of ACD.

PT may remain the gold standard for identification of ACD and the allergen causing the reactions. However, given the general trend towards more molecular assays in identifying disease, it would not be unrealistic to assume that isolation of protein-bound haptens in active ACD lesions may provide the highest sensitivity test to diagnose ACD. Furthermore, it may also be useful in cases where the causative agent cannot be identified through PT alone and may be beneficial in narrowing the selection of antigens a clinician should test. Ideally, more research should be done on this subject so that identification of certain biomarkers on a blood test could provide physicians with a definitive diagnosis of ACD, but the overlap between ACD and other common conditions makes this unlikely.
